# Progress in perceptual research: the case of prosopagnosia

**DOI:** 10.12688/f1000research.18492.1

**Published:** 2019-05-31

**Authors:** Andrea Albonico, Jason Barton

**Affiliations:** 1 Human Vision and Eye Movement Laboratory, Departments of Medicine (Neurology), Ophthalmology and Visual Sciences, Psychology, University of British Columbia, Vancouver, Canada

**Keywords:** face recognition, neuroimaging, diagnosis, rehabilitation, object recognition

## Abstract

Prosopagnosia is an impairment in the ability to recognize faces and can be acquired after a brain lesion or occur as a developmental variant. Studies of prosopagnosia make important contributions to our understanding of face processing and object recognition in the human visual system. We review four areas of advances in the study of this condition in recent years. First are issues surrounding the diagnosis of prosopagnosia, including the development and evaluation of newer tests and proposals for diagnostic criteria, especially for the developmental variant. Second are studies of the structural basis of prosopagnosia, including the application of more advanced neuroimaging techniques in studies of the developmental variant. Third are issues concerning the face specificity of the defect in prosopagnosia, namely whether other object processing is affected to some degree and in particular the status of visual word processing in light of recent predictions from the “many-to-many hypothesis”. Finally, there have been recent rehabilitative trials of perceptual learning applied to larger groups of prosopagnosic subjects that show that face impairments are not immutable in this condition.

The face is a complex structure. It has a complicated three-dimensional shape, a substantial degree of mobility, and structural constraints that make all faces fairly similar; all of these issues present challenges to a perceptual system. Nevertheless, perhaps because of the social importance of faces, humans have developed the ability to recognize faces rapidly and accurately and with seemingly little effort. Indeed, recent estimates are that the typical person can remember and recognize about 5000 faces ^1^.

However, for some people, face recognition is not so easy. Prosopagnosia is a condition marked by the loss of familiarity for faces and the consequent inability to identify people by their faces ^2^. Although prosopagnosic subjects frequently turn to other cues such as voice, hairstyle, or anomalous facial features, these strategies have their limitations; as a result, prosopagnosic subjects still often find social situations stressful, and recent work has shown that they can suffer from anxiety, depression, and social withdrawal ^3, 4^.

Studies of prosopagnosia have a time-honoured place in research on face recognition. Neuropsychological observations have played key roles in the development of cognitive models of face processing ^5^ and pointed to the cerebral substrates of face recognition ^6, 7^. Even in an era when advances in face research are coming from psychophysics, functional neuroimaging, and primate neurophysiology, there are still important contributions from work on prosopagnosia. This has been spurred particularly by the recognition of a developmental variant ^8^. Although acquired prosopagnosia is rare, developmental prosopagnosia appears to be more common but debate on its exact prevalence continues ^9^. Nevertheless, the greater availability of developmental subjects has led to an increase in the number of prosopagnosic studies. In this review, we focus on four areas of recent progress in the fields of acquired and developmental prosopagnosia.

## The diagnosis of prosopagnosia

Uniform definitions are a critical starting point for research into a condition. The core defects in prosopagnosia are the loss of familiarity for previously known faces and the inability to learn to recognize new faces. In the past, this was often shown by tests using famous faces or in case studies by demonstrations that the subject could not recognize friends or family members. However, it is difficult to derive uniform diagnostic criteria from such tests. Familiarity for famous faces is affected by the subject’s age, culture, education, and interests, for example, and carefully matched controls are essential for interpreting the results of such tests. This has led to supplementation of famous face tests by the increasing use of tests that assess short-term familiarity. These show faces in a learning phase and then present these “target” faces along with new “distractor” faces in a test phase in which subjects are asked to indicate which were the faces they had learned. The most well-known examples are the Warrington Recognition Memory Test ^10^ and the Cambridge Face Memory Test ^11^, the latter of which has the desirable feature of testing recognition across changes in pose or lighting. Compared with tests that use famous or personally known faces, tests of short-term familiarity provide limited exposure and lack the semantic and perceptual richness of long experience but have the advantage of uniformity in the degree of learning and testing. For the Cambridge Face Memory Test, there has also been substantial normative work showing good internal consistency (Cronbach’s alpha ranges from 0.83 to 0.89) and no effects of intelligence or the ethnic mix of faces in the subject’s life experience. There is a very modest advantage for women but a more significant effect of age in that accuracy declines for those over the age of 50 ^11– 13^. Also, versions of this test have been developed for use in children ^14^.

There are many other tests of face processing and these were recently reviewed in detail and categorized ^15^. Diagnostic tests can be divided into three main types: (a) tests of face perception, which can include detecting faces in arrays or discriminating or matching simultaneously seen faces; (b) tests of face recognition, such as the tests for short- and long-term familiarity which were discussed above; and (c) tests of face identification, which involve naming or providing other information learned about the person whose face is shown. Prosopagnosic subjects are impaired on both recognition and identification. Performance on tests of face perception can be used to differentiate between prosopagnosic subjects who have an apperceptive variant, in which there is an under-specification of facial structure by perceptual processing, or an associative or amnestic variant, in which the problem is not perception but the ability of perceptual information to access facial memories ^16^. Examples of tests assessing face perception are the Benton Facial Recognition Test ^17^, the Cambridge Face Perception Test ^18^, the Glasgow Face Matching Test ^19^, and the Caledonian Face Test ^20^. Tests of face imagery have also been used to clarify the status of facial memories and diagnose the amnestic variant ^21^.

Self-report questionnaires are becoming more common tools in diagnosing prosopagnosia. They are quick and easy, do not require equipment, do not need to be done in person and hence can be used to screen a large number of subjects, even at a distance. Among those are the Kennerknecht 15-item questionnaire ^22^, the 20-item Prosopagnosia Index ^23^, and the Cambridge Face Memory Questionnaire ^24^. A potential concern is that individuals may have only modest insight into their face recognition abilities ^25, 26^, particularly children ^27^, although some studies suggest that this might not be the case for adults using the Prosopagnosia Index ^28, 29^. This concern might account for the fact that questionnaires may have high reliability but only modest sensitivity and specificity for diagnosing prosopagnosia ^24^. Because of these concerns, some have advocated that questionnaires always be supplemented by objective tests for diagnosis ^9, 24, 30^.

Recent reviews have discussed how to incorporate these various instruments into a diagnostic approach. This may be less of an issue for acquired prosopagnosia, in which the combination of an appropriate lesion on imaging, the subject’s awareness of a change in face recognition after lesion onset, and poor performance on an objective test of face recognition makes the diagnosis plausible. For developmental prosopagnosia, there are no definite structural or genetic markers at present and so its diagnosis still rests solely on behavioural tests. One review pointed out the wide variations between studies in the types of tests, the number of tests, and the statistical cutoffs used ^9^. This creates variable confidence in the diagnosis and introduces heterogeneity that can confound comparisons across groups and studies, an obstacle to scientific progress. As a result, there have been proposals for more uniform diagnostic criteria ^9, 31^. These include (i) subjective difficulty recognizing faces in daily life; (ii) objectively impaired face recognition on at least two tests of face recognition and criteria of at least 2 standard deviations below control means; (iii) intact general perceptual and memory function; and (iv) exclusion of other disorders associated with impaired face recognition, such as autism spectrum disorders.

Although reaching a firm diagnosis of developmental prosopagnosia has its hurdles, a recent study using qualitative methods suggested that screening for it may be possible with a simple list of 16 “hallmark symptoms” from experiences in daily life, which anyone can review ^27^. The utility and sensitivity of this approach need to be explored.

## The neural basis of prosopagnosia

The older literature has shown that lesions of acquired prosopagnosia are bilateral ^6, 7^ or limited to the right hemisphere ^32, 33^, and reports of left-sided lesions alone are rare ^34– 36^. This is consistent with evidence from functional neuroimaging that face processing induces greater activation in the right hemisphere ^37^. The areas involved are the ventral occipito-temporal and fusiform cortex or anterior temporal cortex or both. These anatomic variants may correspond to functional variants ^16^. Individuals with occipito-temporal or fusiform lesions are more likely to have an apperceptive variant ^38^, whereas those with anterior temporal lesions have an amnestic variant along with better perceptual function and more difficulty with face imagery ^39^.

Although by definition subjects with developmental prosopagnosia do not have large visible lesions, the status of their face processing networks can be studied with more subtle neuroimaging techniques, including measures of cortical thickness, the degree of functional activation, and connectivity within the network. The results as they currently stand are not conclusive. There are two main views. One proposes that developmental prosopagnosia is marked by alterations in various regions of the face network, particularly the fusiform gyrus, changes such as reduced cortical thickness or density ^40, 41^, reduced face selectivity of their activation ^40, 42– 44^, local white matter abnormalities on diffusion imaging ^45, 46^, or reduced feedforward connectivity from early visual to occipito-temporal cortex ^47^. The second proposes a disconnection between posterior and anterior regions within the face network ^48, 49^ on the basis of observations of preserved activation of the fusiform and ventral occipito-temporal cortex by faces ^50– 52^ and abnormalities in long white matter tracts that link posterior and anterior temporal cortex ^53, 54^.

Comparisons with other developmental disorders might be informative. Researchers on dyslexia have suggested a model in which a general risk for cortical anomalies is modulated by other genetic and/or environmental factors that determine the location and extent of such anomalies ^55^. The latter determines the specific syndrome and can explain the frequent co-association of developmental disorders. In this regard, we note recent observations of associations between congenital amusia and developmental prosopagnosia ^56, 57^. Along these lines, others have speculated that abnormal neural migration may be responsible for developmental prosopagnosia ^8^.

Does developmental prosopagnosia have a genetic cause? Face recognition abilities show a high degree of heritability in the general population ^58, 59^, and early observations were that developmental prosopagnosia tended to run in families ^59– 63^, possibly with an autosomal dominant pattern of inheritance ^22, 64^. However, most neurodevelopmental disorders are polygenic combinations of allelic variants present in the normal population. Along these lines, a recent study of 24 subjects reported that common single-nucleotide polymorphisms in the oxytocin receptor gene are associated with developmental prosopagnosia ^65^. These preliminary results require replication in larger samples.

## Is prosopagnosia only about faces?

A long-standing controversy is whether the impaired recognition in prosopagnosia is face-specific or affects other object types. This has important theoretical implications for how object recognition is organized in the visual system. The distributed view suggests that object processing is performed by networks of visual regions, and that some of these regions are involved in the perception of several types of stimuli ^66– 68^. The modular view claims that different categories of objects—particularly faces—are processed by distinct dedicated cortical regions ^69– 71^.

Case studies of acquired prosopagnosia have produced mixed results; some reported normal recognition of exemplars of other objects ^72– 82^ and others showed impairments ^80, 81, 83– 88^. A recent major review ^89^ examined 238 cases of developmental prosopagnosia in the literature. The majority of subjects had evidence of impaired object recognition, although a smaller number had reasonable evidence that object recognition was intact, given that they had both good accuracy and normal reaction times on tests. Although the authors concluded that the frequent association of face and object impairments supported a shared mechanism for recognizing faces and other objects ^89^, the challenge for any comprehensive explanation is to account for both frequent associations and occasional dissociations. One of the most useful aspects of this review was the collection of accompanying commentaries ^90– 104^, which suggested both various hypotheses to explain this fact and methodologic limitations in the currently available data that need to be addressed in future work to allow a more definitive set of conclusions to be drawn.

A particular object type deserves comment – namely, words. One of the difficulties in comparing faces and objects is that humans have a great deal of experience and expertise with faces but such expertise cannot be assumed for other object types. Take cars, for example. A recent study found that, as a group, subjects with developmental prosopagnosia tended to score low on the Cambridge Car Recognition Test but that individual scores ranged quite widely, from excellent to poor ^105^. However, not everyone is a car expert and variable expertise could affect recognition performance. In another group of studies, when visual car recognition scores were adjusted for car expertise, as reflected by a subject’s semantic knowledge about cars, subjects with both acquired and developmental prosopagnosia tended to perform worse than expected ^16, 106, 107^.

In literate societies, visual words, in contrast to cars, are a category for which almost all subjects have considerable perceptual expertise. The “many-to-many hypothesis” proposes that face and visual word processing share and compete for neural resources in regions like the fusiform gyrus and that structural constraints cause visual words to be processed more on the left, in proximity to language processing, and faces secondarily to lateralize to the right ^108– 111^. Lateralization is incomplete, though, and functional imaging shows overlap between face- and word-activated voxels ^112^. As a consequence, the hypothesis predicts that prosopagnosia from right lesions should be accompanied by mild reading deficits in the processing of words and that alexia from left lesions should be accompanied by mild face recognition problems ^108^. Whereas one study of three subjects with acquired prosopagnosia did show mild word recognition deficits ^113^, other studies of visual word processing in acquired prosopagnosia from right-sided lesions alone have not found impaired reading ^114, 115^ and the same is true for developmental prosopagnosia ^116– 118^. On the other hand, the type of processing that is performed on words and faces may differ by hemisphere. Although subjects with acquired prosopagnosia from right-sided lesions may read normally, they often have trouble recognizing handwriting or font ^119– 121^, and subjects with alexia may recognize face identity ^122^ but have trouble with lip reading ^119, 123, 124^.

## Can prosopagnosia be treated?

Spontaneous resolution of acquired prosopagnosia is rare ^125– 127^, and developmental prosopagnosia is a lifelong disorder. Hence, means of improving face recognition skills in these populations are of clinical interest. But can it be done? Neuroimaging shows that face processing activates a widely distributed network, including occipito-temporal, superior temporal, anterior temporal, and inferior frontal regions in both hemispheres, though more on the right ^128^. It is highly unlikely that acquired lesions will eliminate all components of this network; furthermore, some studies in developmental prosopagnosia continue to show activation of this network by faces ^50– 52^. The open question is whether surviving components of the face network in a given prosopagnosic subject have any capacity for functional reorganization or modulation that could allow face recognition to improve through a rehabilitative approach ^129^.

Most work has focused on behavioural interventions, although there is one intriguing report of transient improvement of developmental prosopagnosia after intranasal inhalation of oxytocin ^130^. These rehabilitative attempts have been reviewed in detail ^129, 131, 132^. Approaches can be divided into compensatory strategies, which aim to achieve person recognition by circumventing the face processing impairment, and remediation, which aims to improve that impairment. In terms of the process targeted, they can also be divided into those that focus on enhancing mnemonic function, which has been used in a few case studies ^133– 135^, and those that target perceptual function. As examples of the latter, a few older case studies attempted to enhance attention to facial features, though results on face recognition were variable ^134, 136– 138^.

The most significant recent advances have been trials of perceptual learning in groups rather than single cases of prosopagnosia. In one study of 24 subjects with developmental prosopagnosia ^139^, subjects learned over the course of 2 weeks to discriminate distances between facial features, namely the distance between the eyes and eyebrows or between the nose and the mouth. These “spatial relations” can be thought of as indices of the complex geometry of faces, and studies show that some people with prosopagnosia are impaired in perceiving them ^38^. This trial found improved face perception (but only if the test faces had a similar frontal view) and some modest improvements in subjective reports of daily experience with faces. A second study of 10 subjects with acquired prosopagnosia ^132^ used morphed faces to train subjects over the course of 11 weeks to perceive finer and finer differences in facial shape; at the same time, the study introduced irrelevant variations in the expression and viewpoint of the face. In these subjects, compared with a control condition, there was a 21% absolute increase in perceptual sensitivity to facial shape after training, which generalized over new views and expressions. Importantly, there was also a 10% increase for new faces on which subjects had not trained, indicating that subjects were acquiring new skills rather than just learning a set of faces. The effects of training were still evident 3 months later. Although some but not all subjects related anecdotes pointing to improved face recognition in daily life, future studies will require formal evaluation of real-life benefit before such methods are translated to the clinic.

These rehabilitative studies represent a starting point. Although neither training method represents a “cure”, they provide evidence that face processing can be changed in prosopagnosia. They also suggest that there may be individual differences in training potential. Further work is required to determine whether the perceptual gains from learning can be augmented further by better training design or the use of adjunctive methods to promote plasticity during learning.
